# Screening mitochondrial DNA sequence variation as an alternative method for tracking established and outbreak populations of Queensland fruit fly at the species southern range limit

**DOI:** 10.1002/ece3.2783

**Published:** 2017-03-18

**Authors:** Mark J. Blacket, Mali B. Malipatil, Linda Semeraro, Peter S. Gillespie, Bernie C. Dominiak

**Affiliations:** ^1^ Agriculture VictoriaAgriBio, Centre for AgriBioscienceBundooraVictoria 3083Australia; ^2^ School of Applied Systems BiologyLa Trobe UniversityBundooraVictoria 3083Australia; ^3^ Agricultural Scientific Collections UnitDepartment of Primary Industries New South WalesOrange Agricultural InstituteOrangeNSWAustralia; ^4^ Department of Primary Industries New South WalesOrangeNSWAustralia

**Keywords:** *Bactrocera tryoni*, mitochondrial DNA sequences, pest fruit fly, population genetic structure, Queensland fruit fly, southeastern Australia, species border

## Abstract

Understanding the relationship between incursions of insect pests and established populations is critical to implementing effective control. Studies of genetic variation can provide powerful tools to examine potential invasion pathways and longevity of individual pest outbreaks. The major fruit fly pest in eastern Australia, Queensland fruit fly *Bactrocera tryoni* (Froggatt), has been subject to significant long‐term quarantine and population reduction control measures in the major horticulture production areas of southeastern Australia, at the species southern range limit. Previous studies have employed microsatellite markers to estimate gene flow between populations across this region. In this study, we used an independent genetic marker, mitochondrial DNA (mtDNA) sequences, to screen genetic variation in established and adjacent outbreak populations in southeastern Australia. During the study period, favorable environmental conditions resulted in multiple outbreaks, which appeared genetically distinctive and relatively geographically localized, implying minimal dispersal between simultaneous outbreaks. Populations in established regions were found to occur over much larger areas. Screening mtDNA (female) lineages proved to be an effective alternative genetic tool to assist in understanding fruit fly population dynamics and provide another possible molecular method that could now be employed for better understanding of the ecology and evolution of this and other pest species.

## 1 Introduction

The Queensland fruit fly, *Bactrocera tryoni* (Froggatt; Diptera: Tephritidae), a highly polyphagous pest capable of breeding in hundreds of different host fruits (Hancock, Hamacek, Lloyd, & Elson‐Harris, 2000), is one of the most serious Australian insect pests and is responsible for significant economic costs associated with fruit production in eastern Australia (Clarke, Powell, Weldon, & Taylor, 2011). The general biology of *B. tryoni* has been studied for over a century, with a large body of the literature regarding fly outbreaks and associated population control (Clarke et al., 2011; Dominiak, 2012; Dominiak, Daniels, & Mapson, 2011; Dominiak & Ekman, 2013).

### 1.1 *Bactrocera tryoni* distribution and management

**Figure 1:**
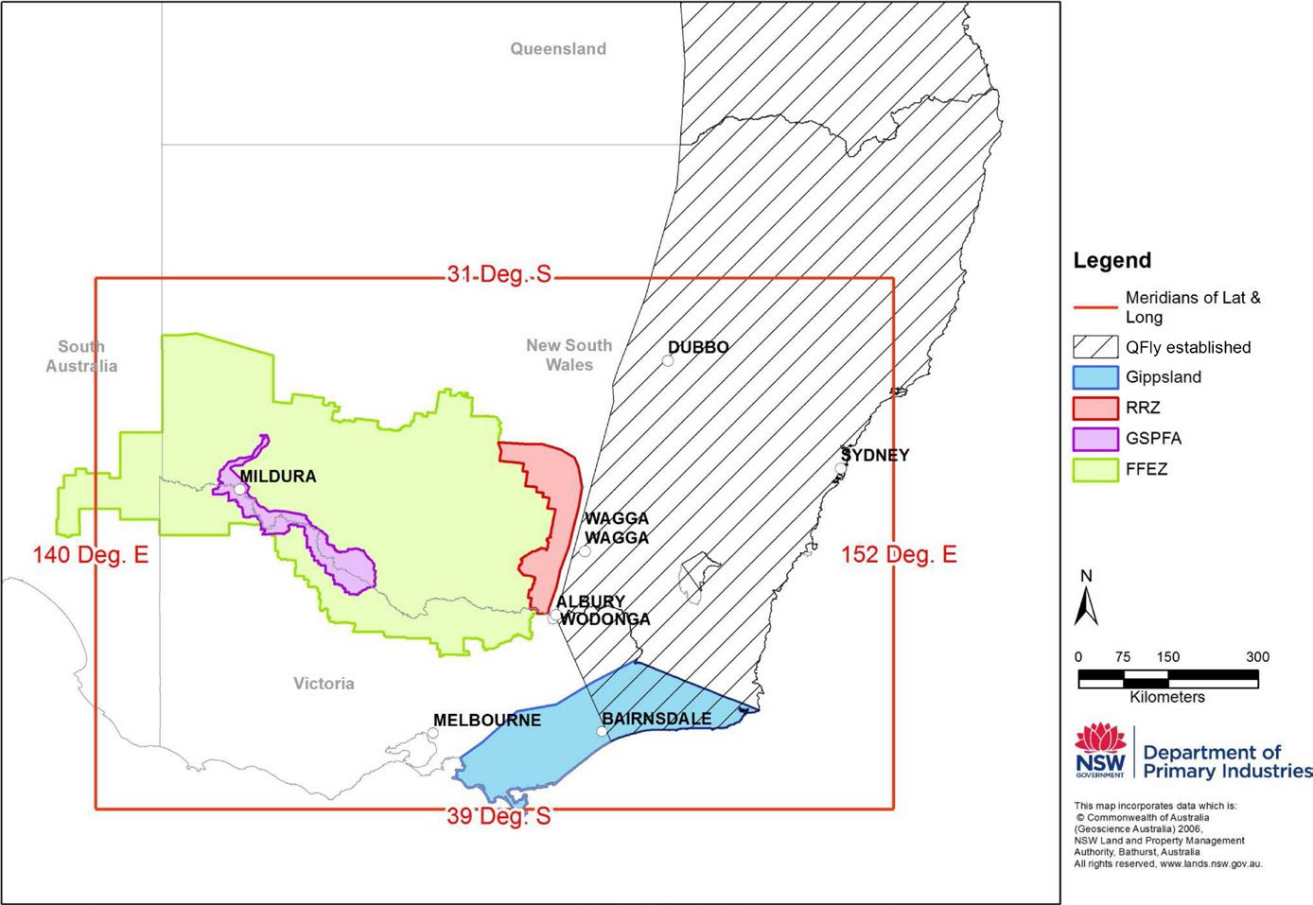
Geographic distribution of *Bactrocera tryoni* at the species range limit in southeastern Australia (adapted from Dominiak & Daniels, 2012), with the boundaries of the fruit fly management control regions mentioned in the text indicated. The red box indicates the area sampled in this study (Victoria and southern New South Wales)

*Bactrocera tryoni* occurs along the entire east coast of Australia from the tropics in Queensland to temperate eastern Victoria (Dominiak & Daniels, 2012). *Bactrocera tryoni* is restricted to Australia (endemic) and some Pacific islands and is of significant trade concern for national and international export of some horticultural products (Plant Health Australia 2016). The history of *B. tryoni* control was recently reviewed by Dominiak and Ekman (2013). Large production areas in southern Australia benefit from market access opportunities and avoided production losses through being free of this significant pest (Clarke et al., 2011). The main horticultural production areas across Victoria, New South Wales, and South Australia in southeastern Australia—were until very recently covered by a large Fruit Fly Exclusion Zone (FFEZ)—developed in 1995 (Figure 1), with procedures to manage the FFEZ and permitted control measures (Dominiak & Daniels, 2012). A second zone of higher quarantine management, the Greater Sunraysia Pest Free Area (GSPFA), was established in 2007 within the FFEZ (Figure 1). The aim of the GSPFA was to optimize international market access for local stone fruit, table grapes, and citrus producers. The GSPFA consists of a long zone following Australia's largest river, the Murray River, while the FFEZ involved a larger area encompassing large areas of semiarid landscape, the latter largely not supporting the survival of *B. tryoni* (Dominiak, Mavi, & Nicol, 2006). A further informal Risk Reduction Zone (RRZ) also existed at the boundary of the FFEZ and established region (Figure 1).

Management within these control zones is based on surveillance (Dominiak, Gilmour, Kerruish, & Whitehead, 2003; Dominiak & Nicol, 2010). When detection exceeds a prescribed threshold (Dominiak et al., 2011), an outbreak is declared and control measures immediately instituted, including spraying and restrictions on the movement of locally grown host produce (Dominiak & Ekman, 2013). However, recently, the pesticides used in cover sprays in some eradication programs have been reviewed and their use patterns are now much more restricted (Dominiak & Ekman, 2013).

### 1.2 Molecular markers for *B. tryoni* surveillance

To achieve the most effective control of the spread of *B. tryoni*, a major concern is tracking fly dispersal, including understanding the origins of new incursions/outbreaks. A large number of nuclear genetic markers (microsatellites) have been developed for *B. tryoni* (Kinnear, Bariana, Sved, & Frommer, 1998; Wang, Yu, Raphael, & Gilchrist, 2003; Zhou, Frommer, Sved, & Gillies, 2003), which have been employed to examine underlying population structure in this species (Cameron, Sved, & Gilchrist, 2010; Gilchrist, Dominiak, Gillespie, & Sved, 2006; Gilchrist & Meats, 2010; Wang et al., 2003; Yu et al., 2001; Chen, Dominiak, & O'Rourke, 2016), including the structure and persistence of *B. tryoni* outbreak populations. Many additional nuclear markers, including thousands of SNPs, were recently characterized from the *B. tryoni* genome (Gilchrist et al., 2014); however, these are yet to be employed to characterize *B. tryoni* populations (Sherwin et al., 2015).

Previous microsatellite studies have indicated the presence of a number of genetic populations in southeastern Australia, including a genetic cline between two populations in the RRZ along the border of the established and managed zones (Gilchrist & Meats, 2010). Recently, the use of microsatellites for management of *B. tryoni* outbreaks has been significantly improved through the optimization of nine loci to work together in a single multiplex PCR (Chen et al., 2016), greatly reducing the labor required to genetically screen specimens. However, the use of microsatellite data for genetic screening does still have some limitations including the following: (1) They can require relatively large sample sizes to define populations (e.g., Gilchrist, Sved, & Meats, 2004); (2) they are also not particularly suited to the cumulative addition of samples, owing to potential differences in allele scoring between different laboratories and genotyping platforms (e.g., Moran, Teel, LaHood, Drake, & Kalinowski, 2006).

An alternative molecular marker, mitochondrial DNA (mtDNA), that is inherited independently from nuclear (microsatellite) markers has previously been employed for species identification of *B. tryoni* (Armstrong & Ball, 2005; Blacket, Semeraro, & Malipatil, 2012; Cameron et al., 2010). MtDNA DNA sequences are ideal for cumulative studies, as they can be unambiguously scored and can be built up into a spatial and temporal “library” as samples become available. MtDNA sequences are also relatively cheap to obtain and simple to analyze, in comparison with nuclear markers such as microsatellites. Despite this, mtDNA sequences have not been widely used for estimating the underlying population structure of *B. tryoni* outbreaks to date. They have, however, recently been employed to determine underlying populations in other fruit fly species (Karsten, van Vuuren, Barnaud, & Terblanche, 2013; Kunprom, Sopaladawan, & Pramual, 2015; Meixner, McPheron, Silva, Gasparich, & Sheppard, 2002; Schutze et al., 2012; Shi, Kerdelhué, & Ye, 2010, 2012). Additional attributes of mtDNA sequences: single copy genes with high levels of genetic variation that are inherited clonally through the maternal (female) parent, make mtDNA markers ideally suited for tracking breeding success of pest lineages (e.g., Blacket, Rice, Semeraro, & Malipatil, 2015).

To date, a variety of different mtDNA loci have been examined (Blacket et al., 2012; Cameron et al., 2010; Morrow et al., 2000; Shearman, Frommer, Morrow, Raphael, & Gilchrist, 2010). However, none of these studies have included a large number of samples of *B. tryoni* from southern Australia at the species natural range limit (Dominiak & Daniels, 2012). Some fruit fly species are known to be limited by environmental conditions (e.g., Royer, Wright, & Hancock, 2016), and southern Australia is believed to be suboptimal for *B. tryoni* (Gilchrist & Meats, 2010; O'Loughlin, 1984), with temperature, availability of moisture and host fruit believed to be the major factors determining the suitability of areas for *B. tryoni* (Clarke et al., 2011; Dominiak et al., 2006). Previous studies suggest that some of the most southerly known established populations, from east Gippsland Victoria (Figure 1), may have adapted to colder environmental conditions (O'Loughlin, 1964).

### 1.3 Objectives

The primary aim of this study was to assess the utility of using DNA sequences from a single mtDNA marker (i.e., haplotypes) to detect the underlying population structure within and between established and outbreak *B. tryoni* populations within different fruit fly management control regions (Figure 1) in southeastern Australia (i.e., spatial variation). We also examined haplotype variation over time (i.e., temporal variation) at a small number of selected sites that were historically subject to different *B. tryoni* control measures.

## 2 Materials and Methods

### 2.1 Samples

#### 2.1.1 Adults

A total of 765 adult specimens from 63 locations were examined in this study (Table 1). All of these specimens were adult male *B. tryoni* collected during 2010 and 2011 (with a small number of additional archived specimens collected in 2008 also examined for the temporal study) from Lynfield traps based with Cuelure by the Victorian and NSW Department of Primary Industries (Agriculture Victoria/NSW DPI) during routine fruit fly surveillance, from across southeastern Australia (Dominiak et al., 2003, Figure 1). The regions sampled included established areas of southern NSW, northern Victoria, and outbreak sites in the FFEZ, the GSPFA, as well as established and outbreak sites from east Gippsland (Table 1). The above a priori management control regions were used in the analyses below as a convenient way of grouping samples that differ in the degree of effort applied to control *B. tryoni* populations (i.e., “Management zone” in Table 1).

**Table 1:** Collection locations for *Bactrocera tryoni* samples, with haplotypes detected at each site

Locality	Code	Latitude	Longitude	*n*	Management zone^a^	Genetic group^b^	Haplotypes detected
Barellan	Bare	−34.28	146.57	10	FFEZ	Other	3, 5, 38, 50, 74, 111
Barooga	Baro	−35.90	145.70	16	FFEZ	Southern FFEZ	2, 4, 18, 108, 109, 110
Beechworth	Beec	−36.35	146.68	5	FFEZ	Southern FFEZ	10, 11, 59
Berrigan	Berr	−35.67	145.82	5	FFEZ	Central FFEZ	6, 18
Cobram East	CobE	−35.98	145.73	7	FFEZ	Other	3, 4, 6, 117, 119, 120
Cobram East	Cobr	−35.92	145.63	11	FFEZ	Other	5, 6, 38, 61, 118
Cobram South	CobS	−35.98	145.60	11	FFEZ	Other	3, 5, 17, 53, 61, 82
Corowa	Coro	−35.98	146.38	19	FFEZ	Southern FFEZ	2, 12, 25
Darlington	Darl	−34.57	145.98	10	FFEZ	Other	4, 26, 54, 84, 85, 122, 123
Deniliquin	Deni	−35.53	144.97	8	FFEZ	Central FFEZ	6, 9, 27
Dookie	Dook	−36.33	145.68	5	FFEZ	NSW/Gippsland	1
Echuca	Echu	−36.13	144.75	6	FFEZ	NSW/Gippsland	1, 11, 16, 66
Glenrowan	Glen	−36.47	146.22	10	FFEZ	Southern FFEZ	2, 3, 25, 32, 84, 135
Goolgowi	Gool	−33.98	145.72	5	FFEZ	NSW/Eastern FFEZ	3, 13, 136, 137
Griffith	Grif	−34.28	146.05	15	FFEZ	NSW/Eastern FFEZ	3, 9, 11, 13, 138
Hillston	Hill	−33.48	145.53	15	FFEZ	Central FFEZ	1, 2, 4, 5, 6, 21, 32, 41, 44, 64, 141, 142, 143, 144
Howlong	Howl	−35.98	146.63	10	FFEZ	NSW/Eastern FFEZ	3, 6, 9, 13, 18, 52
Kyabram	Kyab	−36.32	145.05	9	FFEZ	Other	1, 4, 16, 149
Leeton	Leet	−34.55	146.40	12	FFEZ	Central FFEZ	6, 9, 12, 18, 55, 156
Rutherglen	Ruth	−36.05	146.47	18	FFEZ	Southern FFEZ	2, 3, 11, 13, 25, 26, 34
Shepparton	Shep	−36.37	145.40	5	FFEZ	Other	32, 52, 71
Tocumwal	Tocu	−35.82	145.57	17	FFEZ	Central FFEZ	1, 4, 6, 9, 12, 23, 185, 186, 187, 188
Wahgunyah	Wahg	−36.00	146.42	14	FFEZ	Southern FFEZ	2, 3
Wangaratta	Wang	−36.37	146.32	8	FFEZ	Southern FFEZ	2, 3, 10
Whorouly	Whor	−36.52	146.58	9	FFEZ	NSW/Eastern FFEZ	3, 9
Yanco	Yanc	−34.60	146.42	15	FFEZ	NSW/Eastern FFEZ	1, 3, 9, 18, 55, 192
Yarrawonga	Yarr	−36.02	145.98	10	FFEZ	Southern FFEZ	2, 6, 10, 21, 81
Yenda	Yend	−34.25	146.20	15	FFEZ	NSW/Eastern FFEZ	1, 3, 5, 6, 9, 12, 13, 63, 191
Bairnsdale	Bair	−37.82	147.62	13	Gippsland	NSW/Gippsland	1, 5, 16, 21, 22, 23, 48, 107
Eagle Point	EagP	−37.90	147.68	9	Gippsland	Southern FFEZ	2, 21, 42, 65, 129, 130
Lakes Entrance	LakE	−37.88	147.98	6	Gippsland	Other	3, 21, 32, 75, 150
Marlo	Marl	−37.80	148.53	12	Gippsland	NSW/Gippsland	1, 45, 48, 58, 93, 157, 158, 159
Orbost	Orbo	−37.70	148.45	10	Gippsland	NSW/Gippsland	1, 4, 17, 22, 45, 88, 98
Sale	Sale	−38.12	147.07	11	Gippsland	Other	4, 16, 17, 42, 79
Upper Tambo	Tamb	−37.77	147.87	8	Gippsland	NSW/Gippsland	1, 5, 17, 39, 41, 60, 63
Ardlethan	Ardl	−34.35	146.90	9	NSW	NSW/Gippsland	1, 4, 74, 103, 104, 105
Bathurst	Bath	−33.42	149.58	10	NSW	NSW/Gippsland	1, 3, 4, 16, 17, 20, 22, 58
Coolomon	Cool	−34.82	147.20	8	NSW	NSW/Eastern FFEZ	1, 3, 10, 11, 62
Cootamundra	Coot	−34.63	148.03	10	NSW	NSW/Eastern FFEZ	1, 2, 3, 16, 24
Dubbo	Dubb	−32.25	148.60	22	NSW	NSW/Gippsland	1, 2, 4, 16, 54, 63, 86, 87, 124, 125, 126, 127
Eubalong	Euab	−33.12	146.47	10	NSW	NSW/Gippsland	1, 4, 22, 39, 86, 87, 132, 133
Ganmain	Ganm	−34.80	147.03	10	NSW	NSW/Eastern FFEZ	1, 3, 9, 67, 134
Jemalong	Jema	−33.45	147.80	5	NSW	NSW/Gippsland	1, 17, 54, 89
Lake Cargelligo	LCar	−33.30	146.37	19	NSW	NSW/Eastern FFEZ	1, 4, 11, 24, 62, 92, 93, 151, 152, 153, 154, 155
Mudgee	Mudg	−32.60	149.58	8	NSW	NSW/Gippsland	1, 9, 17, 37, 41, 50, 97, 165
Orange	Oran	−33.28	149.10	17	NSW	NSW/Gippsland	1, 4, 11, 16, 22, 23, 40, 49, 69, 76, 97, 167, 168, 169
Sydney Region	Sydn	−33.87	151.20	12	NSW	Other	5, 17, 27, 49, 59, 98, 181, 182, 183, 184
Wagga Wagga	Wagg	−35.13	147.35	6	NSW	Central FFEZ	6, 12, 13, 18, 22
Wodonga	Wodo	−36.12	146.88	15	NSW	Southern FFEZ	1, 3, 9, 10, 11, 26, 54, 60
West Wyalong	WWya	−33.92	147.20	11	NSW	NSW/Gippsland	1, 4, 50, 80, 95
Barham	Barh	−35.62	144.13	17	GSPFA	Central FFEZ	1, 3, 4, 6, 21, 37, 76
Boundary Bend	Boun	−34.73	143.12	20	GSPFA	NSW/Gippsland	1, 11, 36, 41, 51, 77, 78, 112, 113, 114
Cardross	Card	−34.30	142.13	13	GSPFA	Other	5
Ellerslie	Elle	−33.81	142.04	22	GSPFA	Other	8, 131
Gol Gol	GolG	−34.18	142.22	15	GSPFA	Other	5
Koondrook	Koon	−35.63	144.12	17	GSPFA	Central FFEZ	6, 9, 11, 23, 37
Merbein	Merb	−34.17	142.07	15	GSPFA	Other	29, 40, 95, 162
Mildura	Mild	−34.18	142.17	11	GSPFA	Southern FFEZ	2, 28, 96, 163, 164
Nichols Point	Nich	−34.22	142.22	17	GSPFA	Other	5, 14
Pooncarie	Poon	−33.38	142.57	21	GSPFA	Other	4, 27, 30, 33, 170
Robinvale	Robi	−34.58	142.77	20	GSPFA	Other	4, 31, 32, 36, 99, 171, 172, 173
Speewa	Spee	−35.22	143.52	19	GSPFA	Other	15, 35
Wood Wood	Wood	−35.10	143.33	17	GSPFA	Other	1, 15, 35, 44, 56, 73

FFEZ, Fruit Fly Exclusion Zone; GSPFA, Greater Sunraysia Pest Free Area.

a See Figure 1.

b “Genetic groups” are defined from Figure 4.

#### 2.1.2 Larvae

Additionally, a small number of larval samples were examined in this study. One group of larval samples originated from Queensland (*n* = 12) that were collected by Victorian (Agriculture Victoria) Biosecurity from fruit intercepted at the Melbourne markets. The other larval samples (*n* = 13) were collected from infested fruit grown in the GSPFA during outbreaks, from sites that were also sampled contemporaneously for adult *B. tryoni* (Table 1). Each larval sample tested represented a different larval detection (i.e., a separate infested fruit sample).

### 2.2 DNA extraction, PCR amplification, and DNA sequencing

DNA was extracted from dry fly samples using 5% Chelex (Sambrook, Fritsch, & Manaitis, 1989). The mtDNA locus Cytochrome b (*Cytb*) was chosen for this study as in *B. tryoni*, the amplification conditions have previously been optimized, and it is also known to be highly variable, does not contain indel gaps, or appear to have nuclear copies (numts; Blacket et al., 2012). Laboratory methods for PCR amplification of *Cytb* follow (Blacket et al., 2012). DNA sequencing was performed on an ABI sequencer commercially through Macrogen (Korea). Haplotype sequences of *Cytb* have been deposited in GenBank (accession numbers: KY550463 ‐ KY550654).

### 2.3 Spatial analyses

Spatial autocorrelation was used to detect relationships between genetic and geographic distances within regions, in GenAlEx (Peakall & Smouse, 2006). Geographic distance matrices (km between sites) were estimated in GenAlEx from the latitude and longitude of each site. Genetic distance matrices were calculated for a haploid marker by population in GenAlEx. To confirm the robustness of any significant correlations, multiple distance classes (between 5 and 100 km) were trialed for each spatial autocorrelation analysis (data not shown), and no other statistical corrections were made to account for the large number of spatial autocorrelation comparisons tested.

### 2.4 Genetic analyses

The genetic diversity present at sites and within regions was examined by plotting the number of haplotypes detected at sites, divided by the number of individuals sampled at each site, against the latitude and longitude of each site, in Excel. An analysis of molecular variance (AMOVA) was also conducted in GenAlEx to examine the partitioning of genetic variation within and between regions and sites.

A neighbor‐joining tree of genetic relationships between sites was constructed in Mega 5.1 (Tamura et al., 2011) from a genetic distance matrix based on degree of haplotype sharing between sites (inverse Nei distances) exported from GenAlEx; that is, the tree is based on the frequency of haplotypes in each population, rather than on the actual haplotype DNA sequence differences. Additional detailed population genetic analyses, for example, haplotype networks, were not conducted here as these would be inappropriate for these data given that most sequence differences between haplotypes are likely to have accumulated over long periods of time in the original northern source populations, (which have not been sampled for mtDNA variation to date see below), rather than in situ in southeastern Australia.

### 2.5 Temporal analyses

Finally, the genetic (haplotype) diversity in samples collected from a limited number of sites in Gippsland and the FFEZ in 2008 and 2010 was examined to test for the persistence of mtDNA lineages through time, that is, temporal variation at these sites.

## 3 Results

### 3.1 Overall genetic diversity

A large number of haplotypes (n = 153) were detected from the *B. tryoni* samples in this study (Table 1). The AMOVA indicated that the greatest amount of genetic variation (74%) was present within populations (sites), a substantial amount of variation (24%) was found between populations (sites), while a very small amount (2%) was limited to regional differences. Despite the very low regional distinction indicated above, further analyses were conducted using the a priori management regions defined earlier, as these differ not only in the degree of control measures (e.g., low in established regions compared to high in outbreak areas), but also in their observed levels of observed genetic diversity and levels of gene flow (see results below).

#### 3.1.1 Genetic diversity within sites

**Figure 2:**
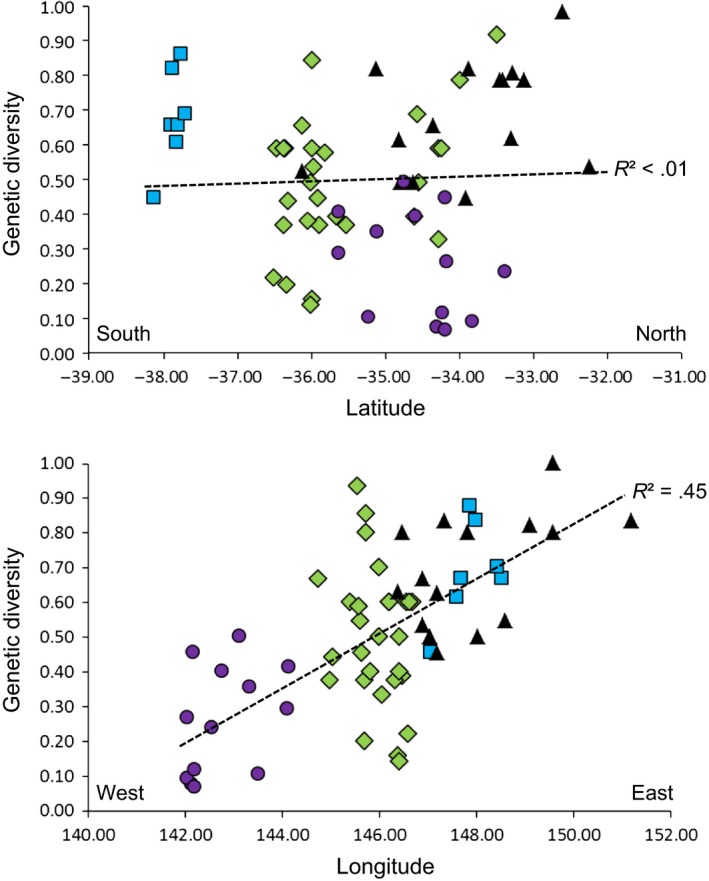
Haplotype diversity of *Bactrocera tryoni* populations compared with latitude and longitude. Geographic regions and management zones are indicated: NSW (triangles), Gippsland (squares), Fruit Fly Exclusion Zone (diamonds), and Greater Sunraysia Pest Free Area (circles), fill colors of these a priori management regions match Figure 1. Linear regressions between genetic diversity with latitude and longitude (all sites analyzed together) are indicated by dashed lines

A comparison of haplotype diversity (i.e., number of alleles/number of samples at a site) compared with site locations was conducted (Figure 2). A strong association (regression analysis) was detected with longitude but not latitude, with each management region also exhibiting different levels of diversity (Figure 2). Sites in the GSPFA possessed low haplotype diversity, FFEZ sites were variable with low to high diversity, while established sites all exhibited high genetic diversity.

### 3.2 Spatial patterns of variation

#### 3.2.1 Spatial autocorrelation within regions

**Figure 3:**
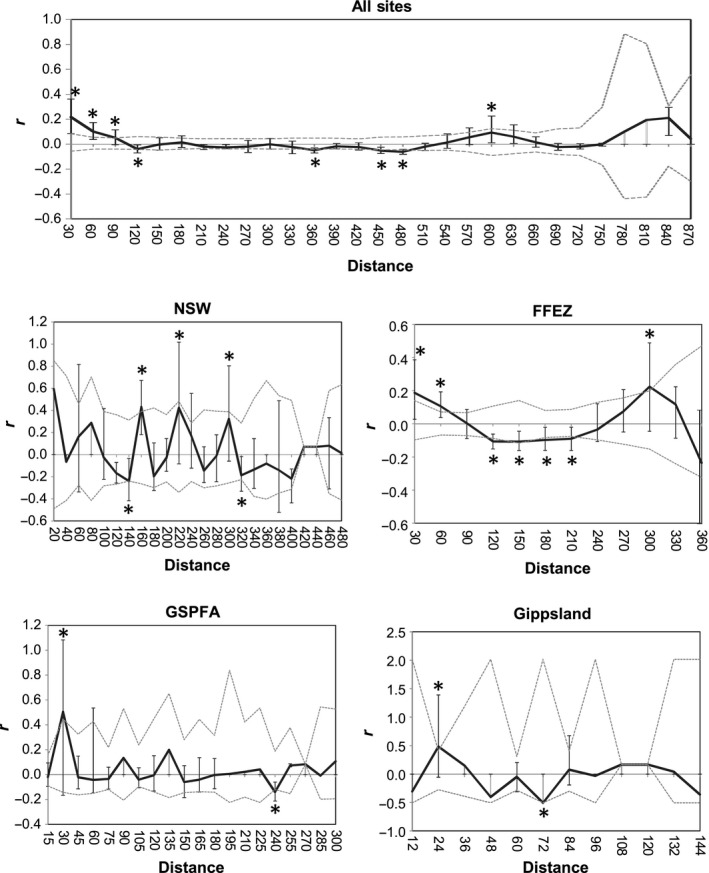
Spatial structure analyses of *Bactrocera tryoni* populations. Statistically significant (*p* < .05) positive and negative correlations between genetic and geographic distances (km) are indicated by an “*”, with dashed lines indicating upper and lower confidence limits. Significant correlations indicate that populations are more similar (+ve correlations), or dissimilar (‐ve correlations) than expected through chance

Spatial autocorrelation analyses were employed to test for possible geographic population structure (Figure 3). Significant positive and negative correlations (*p* < .05) within each region suggested relationships between groups of sites and genetic variation. In a combined analysis of all of the sites together (Figure 3), there were significant positive autocorrelations in all distance classes up to 90 km, suggesting that generally sites that are geographically close are genetically similar. The negative correlations, between 120 and 480 km, suggest multiple populations are present. An additional positive correlation at 600 km indicates that some widely separated sites were also genetically similar (i.e., representing gene flow within a widespread population).

Examining the samples using the a priori management regions revealed additional spatial patterns. Within the FFEZ (Figure 3), sites up to 60 km appear genetically similar, while sites from 120 to 210 km are dissimilar, suggesting multiple populations within this region. Again some distant sites up to 300 km apart are genetically similar, indicating that within the FFEZ, some populations are widely distributed or have common origins perhaps with frequent reintroductions from the same source. Patterns of variation for NSW (Figure 3) show a number of positive and negative correlations, indicative of the presence of multiple genetic populations. Interestingly, geographically adjacent sites in NSW are not necessarily genetically close. This also appears to be the case in Gippsland and the GSPFA (Figure 3), where the only positive correlations are at 24 and 30 km, respectively. Again negative correlations at greater distances indicate the probable presence of multiple genetic populations within Gippsland and the GSPFA.

#### 3.2.2 Genetic relationships between sites

**Figure 4:**
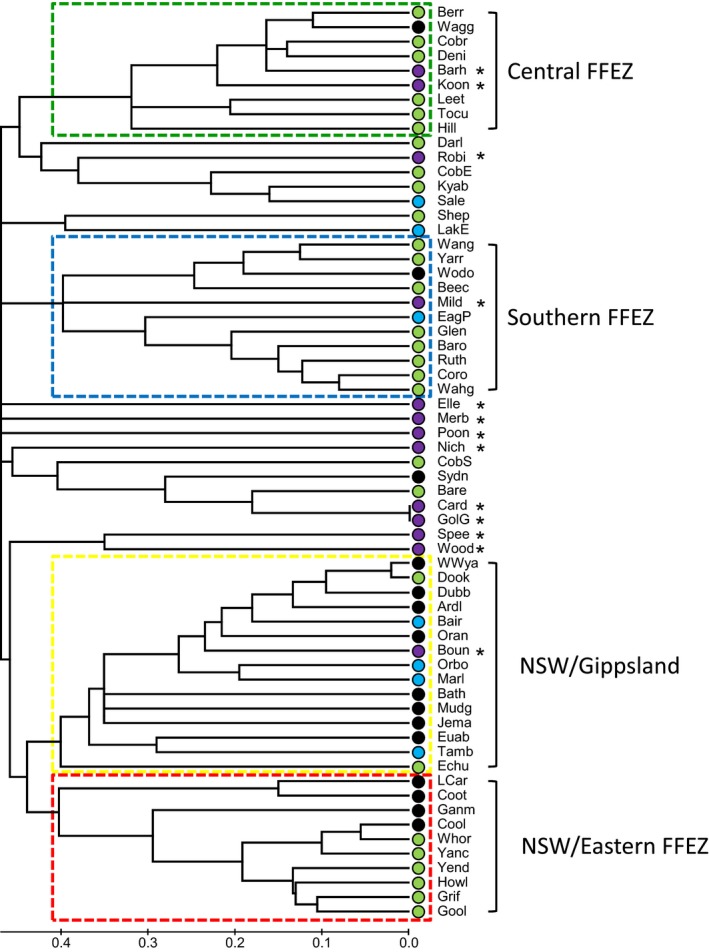
Neighbor‐joining tree of genetic relationships between sites, based on the degree of haplotype sharing (Nei distances), between all *Bactrocera tryoni* collection sites (locality codes from Table 1). Major genetic groups are indicated by colored boxes (dashed lines) and are named after the geographic region in which they generally occur (Figure 5). Greater Sunraysia Pest Free Area sites are highlighted by an “*”, filled circles next to each population indicate a priori management regions from Figure 1

**Figure 5:**
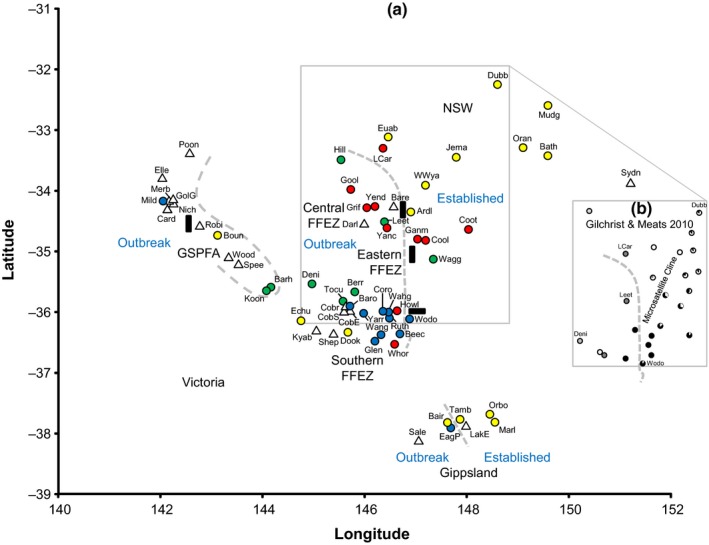
(a) Collection localities of *Bactrocera tryoni* (locality codes from Table 1), indicating regions and management zones, approximate boundaries between zones (dashed lines), and approximate location of long‐term roadblocks (black rectangles). Major genetic groups (from Figure 4) are indicated by circles of different colors (yellow = NSW/Gippsland, red = NSW/Eastern Fruit Fly Exclusion Zone (FFEZ), green = Central FFEZ, blue = Southern FFEZ), triangles represent sites that are not closely related, with each being genetically distinct from both each other and the major groups. (b) Inset showing *B. tryoni* populations found previously using microsatellite genotyping—modified from figure 2 in Gilchrist and Meats (2010)

The analysis of genetic relationships (based on the degree of haplotype sharing) between sites sampled in this study (Figure 4) indicated several key points: (1) A number of genetic populations appear localized to geographic regions in southeastern Australia (as suggested by the spatial autocorrelation results above). Two populations occur almost exclusively within the FFEZ (“Central FFEZ” & “Southern FFEZ”), a further group occurs between the FFEZ and adjacent sites in NSW (“NSW/Eastern FFEZ”), while the final common major group occurs predominantly across the most easterly established sites sampled from NSW to Gippsland (“NSW/Gippsland”). The geographic extent of each of these major genetic groups is illustrated in Figure 5a, and the most common haplotypes detected in each population are listed in Table 2; (2) most established Gippsland sites appear genetically similar to each other and to other sites in NSW (Figures 4 and 5a); (3) most outbreak sites in the GSPFA (indicated as asterisks in Figure 4) are not particularly genetically similar to one another. The sites that are genetically similar are geographically directly adjacent to one another, that is, Gol Gol/Cardross, Barham/Koondrook, and Speewa/Wood Wood. These results suggest multiple GSPFA outbreak sources and extremely limited gene flow between outbreaks.

**Table 2:** Common *Bactrocera tryoni* haplotypes associated with geographic regions

Genetic Group	Group *n*	Common haplotypes (frequency)	Combined frequency of common haplotypes
NSW/Gippsland	166	1 (0.27), 4 (0.05)	0.33
NSW/Eastern FFEZ	116	3 (0.31), 13 (0.11), 1 (0.09), 24 (0.08), 9 (0.06)	0.65
Central FFEZ	97	6 (0.29), 9 (0.11), 23 (0.07), 4 (0.06), 12 (0.05), 37 (0.05)	0.64
Southern FFEZ	135	2 (0.36), 10 (0.10), 3 (0.07), 12 (0.05)	0.59
Other	251	5 (0.15), 8 (0.08), 4 (0.07), 14 (0.06), 15 (0.06)	0.42
Total	765		Average: 0.52

Geographic groups have been selected based on genetic similarities indicated in Figure 4. Only haplotypes that account for more than 5% individuals within each group are listed here. FFEZ, Fruit Fly Exclusion Zone.

#### 3.2.3 Larval samples

Almost half of the haplotypes detected from the twelve Queensland samples were unique (*n* = 5, Haps 102, 106, 115, 116, 145), while the rest represented shared widespread (*n* = 2, Haps 4, 5) or localized (*n* = 5, Haps 28, 50, 52, 60, 81) haplotypes present at southern *B. tryoni* sites tested in this study. Interestingly, while the number of Queensland larvae sampled here is low, many of the shared haplotypes occurred at outbreak FFEZ or GSPFA sites (Table 1). The larval samples collected from the GSPFA were found to possess haplotypes that were common in adults tested from the same sites; that is, larvae represented the same mtDNA lineages as adults captured at these sites, confirming that larval samples from these GSPFA sites were most likely the result of adult flies breeding within the GSPFA during the study period.

### 3.3 Temporal patterns of variation

**Figure 6:**
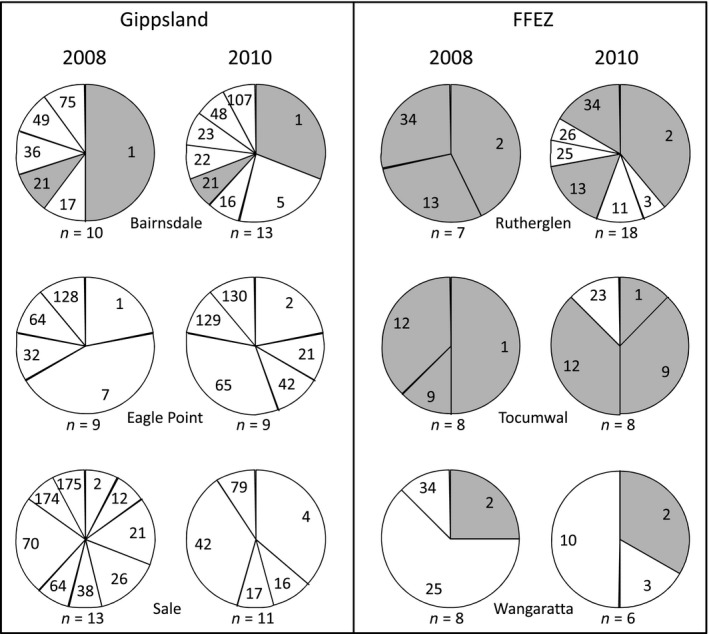
Temporal variation in *Bactrocera tryoni* haplotypes at outbreak sites in Gippsland and the Fruit Fly Exclusion Zone. Samples were collected in 2008 and 2010 from each site; haplotypes that were present at a site in both years are indicated by gray shading

Other indirect evidence for adult *B. tryoni* breeding at outbreak sites is provided by the temporal samples examined from Gippsland and the FFEZ (Figure 6). Despite the relatively low numbers of individuals sampled, all FFEZ sites tested here possessed haplotypes common between the two sampling periods (shaded gray in Figure 6). The Gippsland sites were more variable with an indication of persistence of haplotypes at sites over time only at Bairnsdale, on the border of the established and outbreak zones (Figures 1 and 5). Bairnsdale possessed some haplotypes (Hap 1 and Hap 21) across years (Figure 6), which are both common in Gippsland (Table 1). The presence of the same haplotypes between years at outbreak sites might indicate persistent recolonization from the same source population, or could be due to persistent residual populations at these sites. The latter possibility appears more likely when outbreaks are present continuously, that is, over a number of consecutive years, given that *B. tryoni* are known to have more than one generation a year (Clarke et al., 2011; O'Loughlin, 1984). The lack of shared haplotypes at the other two Gippsland sites (Eagle Point and Sale) appears to indicate nonpersistence of populations and probably different outbreak sources between the years sampled.

## 4 Discussion

### 4.1 Comparison of mtDNA haplotype variation with previous molecular data

Compared with previous multilocus nuclear microsatellite marker studies of *B. tryoni* populations (Chen et al., 2016; Gilchrist & Meats, 2010; Gilchrist et al., 2004, 2006), mtDNA provides information from only a single locus, which has a clonal–maternal mode of inheritance. Differences between these two sets of markers could result in divergent patterns of population genetic variation being detected. For example, all of the samples in the current study were males, so we were unable to examine whether there were sex‐specific differences in dispersal, whereas microsatellites would provide genetic information from both parents for each sample. Microsatellites are also known to evolve at a very fast rate and could potentially be more sensitive population markers than mtDNA. However, in our study, we detected a large amount of mtDNA genetic variation and have shown that screening mtDNA lineages at multiple sites and times does provide an extremely useful tool for helping to understand *B. tryoni* population dynamics, allowing the extent of gene flow to be estimated across regions as well as detecting a number of genetically distinctive outbreak populations. Despite our study's relatively limited population sample sizes (*n* = 5 to 22, Table 1) and simple analyses, we found highly congruent results between the mtDNA data presented here and the previous results from nuclear microsatellite markers, with very similar resolution of underlying *B. tryoni* population genetic structure observed between studies.

Similarities with previous studies include the following: (1) the large geographic scale of widespread established southeastern Australian populations (Figure 5a; Gilchrist et al., 2006); (2) the presence of multiple populations along the border of the FFEZ and NSW, including a similar pattern across the region boundary (Figure 5a) to the population cline previously detected (Figure 5b; Chen et al., 2016; Gilchrist & Meats, 2010; Gilchrist et al., 2006); (3) multiple populations detected within the FFEZ, which cross the border between the FFEZ and the RRZ (Figure 5a; Chen et al., 2016; Gilchrist & Meats, 2010; Gilchrist et al., 2006); (4) outbreak populations appear to have multiple origins, with greatly reduced gene flow in the control regions compared with established regions (Figure 2; Gilchrist & Meats, 2010; Gilchrist et al., 2004, 2006); (5) many of the southeastern Australian sites sampled in both sets of studies appear genetically distinct; for example, the Sydney region was found to be dissimilar to inland NSW for mtDNA (our study) and microsatellites (Gilchrist et al., 2006).

### 4.2 Patterns of mtDNA variation

Overall the spatial analyses of southern *B. tryoni* populations suggest a degree of localization of genetic variation within a number of outbreaks (Table 1, Figure 5a). Examinations of the most common mtDNA haplotypes within each area show that there is a different “suite” of common haplotypes within each, with a single dominant haplotype accounting for almost a third of all haplotypes in these populations (Table 2). The degree of haplotype localization appeared strongest in the GSPFA, moderate in the FFEZ, and least in the established region (Figure 2). This pattern is probably due to limited initial incursions with persistence (i.e., breeding) of specific *B. tryoni* lineages (represented here by different mtDNA haplotypes) within each area. These results indicate that gene flow is greatly reduced in GSPFA outbreak populations. The patterns of diversity between sites observed here appear to be at least partially explained by the different status of *B. tryoni* in each region tested: *B. tryoni* is established through eastern Australia, including east Gippsland (the most southerly sites sampled here); however, all westerly populations of *B. tryoni* examined were the result of outbreaks, with the degree of *B. tryoni* control measures being greatest in the GSPFA.

Movement of *B. tryoni* between sites is described as jump diffusion, that is, the human‐assisted long‐distance movements followed by local diffusion through natural insect flight (Sadler, Florec, White, & Dominiak, 2011). The natural dispersal ability of *B. tryoni* is generally less than one kilometer (Dominiak, 2012), while *B. tryoni* larvae are known to be carried in infested fruit (Dominiak & Coombes, 2009; Dominiak & Daniels, 2012; Dominiak, Rafferty, & Barchia, 1998). Therefore, *B. tryoni* are not believed to naturally disperse or diffuse from the established areas. Size and connectivity between towns may have a large effect on the degree of movement of *B. tryoni*. Dubbo is the largest inland town in NSW and is also situated along the major north–south transport route (Newell Highway) between Australia's capital cities of Brisbane in Queensland (where *B. tryoni* is established) and Melbourne in Victoria (which is *B. tryoni* free). Genetically, Dubbo appears similar to other nearby towns (e.g., Jemalong and Ardlethan), as well as distant sites in Victoria (Figures 4 and 5). Wagga Wagga is the second largest inland town in NSW but is more isolated, not being on a major throughway, although it is on a road system of east–west traffic from Sydney (*B. tryoni* established) into the eastern FFEZ and subsequently to the GSPFA and South Australia (the latter three areas usually being *B. tryoni* free). Interestingly, Wagga Wagga appears more similar to some FFEZ sites, more closely matching sites from the “Central FFEZ” (Figures 4 and 5) than to other nearby towns in NSW. Albury/Wodonga is also a large town situated on the major north–south transport route taking traffic from Sydney (*B. tryoni* established) to Melbourne (*B. tryoni* free), which appear to match the adjacent “Southern FFEZ” sites (Figures 4 and 5), even though road traffic largely does not enter the FFEZ.

Ours is the first genetic study to examine the most southerly *B. tryoni* populations, including samples obtained from Gippsland (Figure 1, Table 1). These southern established populations, whose existence has been known of for at least fifty years, may be adapted to local conditions (O'Loughlin, 1964). In our analyses, Gippsland flies did not appear distinctive overall from other established Victorian or NSW sites (Figures 4 and 5a). However, interestingly Gippsland sites did possess a large number of haplotypes (*n* = 15, Haps 42, 45, 48, 58, 65, 75, 79, 88, 107, 129, 130, 150, 157, 158, 159) that were not found elsewhere in our study. It would be useful in future studies to sample a greater range of *B tryoni* genetic diversity from a greater part of the species range, including sites from the northern parts of the species range, for comparison with the data presented here.

### 4.3 Persistence of populations

At the time of our study, outbreaks in some parts of the southeastern FFEZ had been continuous for more than five years. This region appears to show continuous gene flow over relatively large distances (Figures 2, 3, and 5). Most outbreaks in other parts of the FFEZ and GSPFA were only declared during the study period, and these sites appear to generally exhibit variable (rather than continuous) gene flow. The persistence of some populations within parts of the FFEZ was also supported by our limited examination of temporal variation (Figure 6).

If there was a widespread low‐level resident population within areas of the GSPFA prior to the study period, gene flow between sites would be expected to result in some shared common haplotypes across the GSPFA. This does not appear to be the case, and overall genetic patterns appear to indicate that over the study period, there were many independent introductions of *B. tryoni* into the GSPFA from multiple sources. Furthermore, these introductions appeared to show very limited dispersal between GSPFA sites.

### 4.4 Pest management implications

Florec, Sler, White, and Dominiak (2013) claimed that random vehicle inspections were the most cost‐efficient strategy for maintaining a regional *B. tryoni* freedom. If there is no traveler awareness campaign and no vehicle inspections, about 18% of the traveling public carry fruit (Dominiak et al., 1998), some of which is infested, with these fruit movements creating the long‐distance jump dispersal. Historically, there has been an active random vehicle inspection (see Figure 5a) and a community awareness program at the eastern border of the FFEZ in an attempt to minimize these jump dispersals (Dominiak & Coombes, 2009, 2010). Our results indicate that these activities were not entirely successful, especially within the RRZ. A similar but smaller program was also run on the eastern border of the GSPFA (see Figure 5a). Our results do indicate a degree of effective control of *B. tryoni* movement between many geographically close sites, with numerous adjacent sites in the FFEZ and GSPFA being genetically dissimilar, that is, not from the same outbreak source.

Historically, the management of *B. tryoni* in the FFEZ and GSPFA has relied on insecticide cover sprays; however, the use of some pesticides has been recently restricted (Dominiak & Ekman, 2013). Fruit fly management has become more challenging, with growers now required to follow an area‐wide management approach (Florec et al., 2013; Lloyd et al., 2010). Following the wettest two‐year period on record in the FFEZ (Webb, 2012), eradication of more than one hundred outbreaks became technically unfeasible and economically unsustainable. Legislation underpinning regulation of host produce was withdrawn for the NSW portion of the FFEZ in 2013. The challenges in *B. tryoni* management experienced in the FFEZ since 1996 will now be transferred to the GSPFA. The reduction in control measures since the time our study samples were collected has now likely resulted in the genetic patterns of the GSPFA becoming similar to those observed from the FFEZ. Consequently, the genetic patterns described in our study for GSPFA are likely to be pushed further west into South Australia, an area still under very strict *B. tryoni* control measures. South Australia maintains a stronger vehicle inspection and regulatory program than that implemented for the FFEZ or GSPFA and incursions are less likely in South Australia, compared to the FFEZ and GSPFA. However, any reduction in the current vehicle inspection program on vehicular traffic entering South Australia will increase the risk of incursion (Florec et al., 2013).

Our study provides a snapshot of a particular point in time, when *B. tryoni* was in the process of becoming established at the southernmost extremity of the species geographic range. This study illustrates how the molecular monitoring methods employed here, to better understand pest dispersal, could be applied to other pests in production areas that are trying to develop pest‐free places of production, or areas of low pest prevalence (Dominiak et al., 2015), or in emergency management of recently incurring pests (e.g., Blacket et al., 2015).

## 5 Conclusions

Overall, it appears that screening mtDNA (female) lineages does provide an extremely useful alternative tool for helping to understand *B. tryoni* population dynamics and determining possible sources of outbreaks. In this study, the extent of gene flow was estimated across regions and a number of distinctive populations were detected within the FFEZ and GSPFA, with some genetic lineages appearing to be being maintained (i.e., breeding), while others appeared to have not yet become widespread and persistent at the time of the study.

We provide baseline information on a number of new introductions of *B. tryoni* at the southern extremity of the species range, that could now be built upon, screening additional sites to include a greater part of the range of *B. tryoni* (i.e., sampling a larger part of the overall *B. tryoni* genetic diversity) as well as examining the same sites over time. It would be particularly beneficial to revisit the sites sampled in our study to observe the genetic changes that have occurred since control measures were reduced in the FFEZ and GSPFA.

Future DNA sequencing studies will now be relatively easier to implement using newly available next‐generation high‐throughput amplicon sequencing technologies (McCormack, Hird, Zellmer, Carstens, & Brumfield, 2013). However, it should be noted that many of the haplotypes detected in our study differed by a single base in 710 bp of mtDNA sequence, so smaller amplicons would not allow detection of the same level of population differentiation as found here. We have shown that screening mtDNA haplotype variation is a powerful tool to be added to the suite of other genetic techniques that are currently available, that could be applied to monitoring *B. tryoni* and other similar pests (e.g., other *Bactrocera* sp.) to provide evidence for developing phytosanitary measures for domestic and international markets.

## Conflict of Interest

All of the authors declare they have no conflict of interest regarding this research.

## Data Archiving

Sequence data (i.e., mitochondrial haplotypes) has been submitted to GenBank: accession numbers KY550463 ‐ KY550654.
